# The GAPP Aggressivity Score Correlates with Total Enriched Somatic Variant Burden in Sporadic Pheochromocytoma—A Pilot Study

**DOI:** 10.3390/cancers18121983

**Published:** 2026-06-18

**Authors:** Reut Halperin, Gil Goldinger, Eddie Fridman, Naama Peshes Yaloz, Amit Tirosh, Gadi Shlomai

**Affiliations:** 1 ENTIRE—Endocrine Neoplasia Translational Research Center, The Chaim Sheba Medical Center, Tel Hashomer, Ramat Gan 5266202, Israelnaama.peshesyaloz@sheba.health.gov.il (N.P.Y.); amit.tirosh@sheba.health.gov.il (A.T.); 2 Gray Faculty of Medical and Health Sciences, Tel Aviv University, Tel Aviv 6997801, Israel; gil.goldinger@sheba.health.gov.il (G.G.); eddie.fridman@sheba.health.gov.il (E.F.); 3 Institute of Pathology, The Chaim Sheba Medical Center, Tel Hashomer, Ramat Gan 5266202, Israel; 4 Division of Endocrinology, Diabetes, and Metabolism, The Chaim Sheba Medical Center, Tel Hashomer, Ramat Gan 5266202, Israel

**Keywords:** pheochromocytoma, risk, GAPP, PASS

## Abstract

Background: Pheochromocytomas and paragangliomas (PPGLs) can be metastatic in up to 30% of cases. To assess this risk, scoring systems like PASS and GAPP were developed, yet it is unclear whether these scores correlate with somatic genetic alterations. Methods: A retrospective pilot study. Twenty patients with sporadic pheochromocytoma, stratified into high- and low-risk groups based on GAPP and PASS scores. Somatic variant burden, derived from whole-exome sequencing, and clinical variables were compared between groups. Results: Six patients (30.0%) comprised the high-risk group; all had PASS scores >4 and GAPP scores indicating moderately differentiated PPGLs. Compared with the low-risk group, the high-risk group had larger tumors (4.7 vs. 3.4 cm, *p* = 0.03) and non-significant association with higher diastolic blood pressure (90 vs. 79 mmHg, *p* = 0.09), normetanephrine (8.5 vs. 2.3 X upper limit of normal [ULN], *p* = 0.08), and metanephrine (22.9 vs. 9.25 X ULN, *p* = 0.09) levels. The high-risk group also demonstrated a higher somatic variant burden, particularly for truncating variants (32 vs. 26, *p* = 0.04). As a continuous variable, only the GAPP score, not PASS score, showed a significant positive correlation with variant burden, observed for both non-missense (*r* = 0.57, *p* = 0.009) and frameshift (*r* = 0.47, *p* = 0.04) variants. Conclusions: Higher pathological severity scores are associated with increased rates of severe molecular somatic variants. The stronger link between GAPP score and genetic alteration rates suggests it may better reflect the genomic complexity of sporadic PPGLs.

## Simple Summary

Pheochromocytomas and paragangliomas (PPGLs) are rare tumors that, in 30% of cases, can metastasize. To predict tumor potential aggressiveness, the PASS and GAPP scoring systems are used to assess how the tumor appears under a microscope. This pilot study examined 20 patients with sporadic PPGL and classified them into high- and low-risk groups based on these scores. The high-risk group had larger tumors and showed signs of higher levels of adrenaline derivatives in the urine, which are linked to tumor activity. They also had more genetic mutations, especially ones that disrupt normal gene function. Interestingly, the GAPP score was better at predicting the number of genetic mutations than PASS. This suggests the possibility that GAPP may be a more accurate tool for understanding the complexity and potential risk of these tumors, helping clinicians tailor treatment plans.

## 1. Introduction

Pheochromocytoma and paraganglioma (PPGL) are rare neuroendocrine tumors that arise from the adrenal medulla or sympathetic and parasympathetic ganglia. Approximately 30–40% of PPGLs develop in association with a hereditary PPGL-related syndrome, and an additional 40–50% due to somatic genetic alterations [1,2]. Recurrent or metastatic disease occurs in 15–30% of patients with a higher rate in those with hereditary disease [3,4]. Risk factors associated with recurrent or metastatic disease include carrying a germline pathogenic variant in a succinate dehydrogenase (SDH) cluster gene (especially *SDHB*), younger age, elevated levels of methoxytyramine and normetanephrine, tumor location, and tumor size [5,6,7,8].

Two major histopathological scoring systems were developed to predict the risk of recurrence or metastases of PPGLs; the pheochromocytoma of the adrenal gland scaled score (PASS) and the grading system for adrenal pheochromocytoma and paraganglioma (GAPP) (Supplementary Table S1) [9,10]. The PASS score was developed using 50 metastatic and 50 non-metastatic pheochromocytomas, and the GAPP score was developed using 40 metastatic and 123 non-metastatic pheochromocytomas. Several studies have aimed to validate each score individually as well as to compare their ability to predict recurrent or metastatic PPGL, yet neither score is currently regarded as a gold standard [11]. Furthermore, data regarding the association of these scoring systems with somatic genetic alterations are limited [12]. Taken together, findings from previous studies suggest that there is a need for additional clinical and somatic genetic parameters that might aid in strengthening the predictive power of the PASS and GAPP scoring systems, especially in the absence of germline pathogenic variants in the *SDHB* gene.

The aim of this study was to unravel a possible association between the genetic somatic landscape of sporadic PPGL and histopathological scoring systems. Such an association might provide insight into the mechanisms leading to the development of recurrent and metastatic sporadic PPGLs.

## 2. Material and Methods

The cohort consisted of twenty patients diagnosed with pheochromocytoma between 2015 and 2025 at Sheba Medical Center. Exclusion criteria were a known hereditary PPGL-related syndrome (including SDH-related syndromes, VHL disease, MEN2, and NF1). We chose sporadic cases to assess the ability of GAPP/PASS and somatic genetic analysis to predict PPGL severity in the sporadic group, avoiding bias from highly aggressive genetic syndromes, such as *SDHB*-related PPGLs. Fourteen patients underwent germline genetic testing (whole-exome sequencing), focusing on genes known or suspected to confer hereditary risk for PPGL: *SDHA*, *SDHAF2*, *SDHB*, *SDHC*, *SDHD*, *TMEM127*, *VHL*, *EGLN1*, *EGLN2*, *FH*, *KIF1B*, *MEN1*, *MAX*, *NF1*, *UBC*, *MDH2*, *SLC25A11*, *SUCLG2*, *REXO2*, *DLST*, *DNMT3A*, *GOT2*, *ELOB*, *CUL2*, *RBX1*, *ELOC*, and *RET* [1,2,13,14,15], with no PPGL-related alterations. An additional six patients did not have any personal or family history suspicious of a PPGL-related syndrome. All patients underwent surgical treatment, and tumor slides were evaluated for PASS and GAPP scores by two independent pathologists. In addition, we gathered clinical data including demographics, biochemical assessments (urinary metanephrine and normetanephrine levels, reported as times elevated above the upper limit of normal [ULN]), imaging (tumor size as reported in computerized tomography or magnetic resonance imaging reports), and outcomes (recurrence, mortality). We categorized patients into high-risk and low-risk of recurrence according to PASS and GAPP scores’ cutoffs (Supplementary Table S1).

### 2.1. Histological Assessment

The slides from the cohort cases were retrieved from the Pathology Institute Laboratory repository at Sheba Medical Center. All slides underwent histopathological reassessment and representative slides are presented in Supplementary Figure S1. Two independent pathologists, blinded to clinical outcomes, evaluated PASS and GAPP scores using established published criteria. Disputed cases were re-evaluated by a third independent pathologist for the final result. PASS scores were categorized as <4 and ≥4, aligning with the recognized cut-off point for metastatic disease risk. GAPP scores were classified into three groups: 0–2 indicating well-differentiated (WD), 3–6 indicating moderately differentiated (MD), and 7–10 indicating poorly differentiated (PD), following the published cut-off points for risk stratification. The immunohistochemical staining results were documented and integrated into the dataset.

### 2.2. Whole-Exome Sequencing (WES) of PPGL Samples

Genomic DNA was extracted from 20 formalin-fixed paraffin-embedded (FFPE) PPGL tumor specimens using the QIAamp DNA FFPE Advanced UNG Kit (QIAGEN, Venlo, The Netherlands, #56704) according to the manufacturer’s protocol. Briefly, FFPE blocks (~4 mm^2^ area) were sectioned into 5–10 μm thickness and incubated with deparaffinization solution at 56 °C for 3 min. Samples were subsequently incubated with FTB buffer, proteinase K, and RNase-free water for 1 h at 56 °C and 1000 rpm, followed by 1 h at 90 °C. After discarding the upper phase, UNG enzyme was added, and the samples were incubated at 50 °C for 2 min. Next, RNase A was added, followed by a 15-min incubation with proteinase K at 65 °C and 450 rpm. A mixture of 500 μL AL buffer and absolute ethanol (1:1) was then added to the lysate and loaded onto the extraction column. Finally, the DNA was eluted in 20 μL buffer. Whole-exome libraries were prepared using standard capture protocols and sequenced on the Element AVITI platform to generate paired-end reads (2 × 150 bp). Raw sequencing reads were assessed for quality using FastQC (v0.11.9). Adapter sequences and low-quality bases were trimmed using Trimmomatic (v0.39), retaining reads with Phred quality scores >20 and a minimum length of 36 bp. High-quality reads were aligned to the human reference genome (GRCh38/hg38) using BWA-MEM (v0.7.17), followed by duplicate marking, base quality score recalibration, and indel realignment according to the GATK Best Practices pipeline (GATK v4.1.7.0). Variant calling was performed using GATK HaplotypeCaller, and variants were filtered based on recommended hard-filtering thresholds. Functional annotation of single nucleotide variants (SNVs) and indels was performed with Funcotator (v1.7.0) against the GENCODE and dbSNP reference databases.

Median target depth across the 20 tumors was 104× (range 40–124×); the median fraction of variant sites covered at ≥30× was 96.3%, at ≥50× was 89.4%, and at ≥100× was 54.2% (Supplementary Figure S2). Variant-allele-frequency (VAF) distributions are now reported per sample (median, modal, fraction VAF ≥ 0.10.

Tumor purity was estimated per sample by fitting a Gaussian-mixture model (mclust, G = 1–3) to the somatic VAF distribution and taking twice the mean of the highest-VAF clonal-heterozygous component within the 0.10–0.55 window. Median estimated purity was 0.91 (range 0.78–0.98). FFPE-related C > T deamination was quantified per sample by fitting the COSMIC v3.3 SBS catalogue with MutationalPatterns (10.1186/s13073-018-0539-0); per-sample SBS30 contribution is reported in Supplementary Figure S3. A VAF-threshold sensitivity analysis confirms that the GAPP–burden correlation is robust to the VAF cutoff (VAF ≥ 0.05, ≥0.10, ≥0.20). With no matched germline, each filter targets one of the two main sources of false-positive somatic calls in tumor-only FFPE exomes, deliberately favoring specificity. (i) Removing variants that recur at identical coordinates across samples, plus a ≥5 alternate/≥5 reference read floor, suppresses FFPE/sequencing artifacts, which, unlike independent somatic events in low-burden PPGL, tend to recur at fixed positions. (ii) The stringent gnomAD AF < 1 × 10^−5^ cut-off depletes residual germline variants, justified because tumor-only data demand aggressive germline removal and true PPGL drivers are private and rare. (iii) Excluding recurrently mismapped “dirty” genes removes technical noise. Thus, we effectively removed common germline polymorphisms. This stringent threshold was applied specifically because our study analyzed tumor tissue without matched germline samples, to allow identification of rare variants that will truly capture driver pathogenic variants. Given the rarity of PPGL, any variant with higher prevalence is unlikely to represent a true pathogenic variant. We thus implemented stringent QC filtering to exclude artifact variants arising from low-quality DNA in FFPE samples and to minimize the inclusion of germline polymorphisms that could be misinterpreted as somatic events. Variants in frequently mutated, predominantly passenger genes were excluded to reduce noise from highly mutable regions. The resulting filtered variant set was used for downstream visualization and analyses. We analyzed the frequency of missense, in-frame insertions and deletions, and putative or enriched somatic variant set (ESV). ESVs were defined as somatic variants predicted to truncate the encoded protein, including frameshift insertions, frameshift deletions, nonsense (stop-gain) mutations, and splice site variants.

### 2.3. Statistical Analysis

Continuous variables are presented as median (interquartile range) and compared using the Wilcoxon test, and categorical variables are presented as n (%) and compared using Chi-square test. Correlation analysis was based on the Pearson correlation coefficient. To compare the correlation strength between PASS and GAPP and the somatic variant load, we employed tests for overlapping dependent correlations using the cocor package in Rstudio, version 1.1-4, which accounts for the fact that both correlations share a common variable (variant count). Confidence intervals for the difference between correlations were derived using Zou’s [16] method. Forest plots were generated to display the correlation difference: Δr = r(GAPP score, variants count) − r(PASS score, variants count), with 95% confidence intervals.

## 3. Results

**Figure 1:**
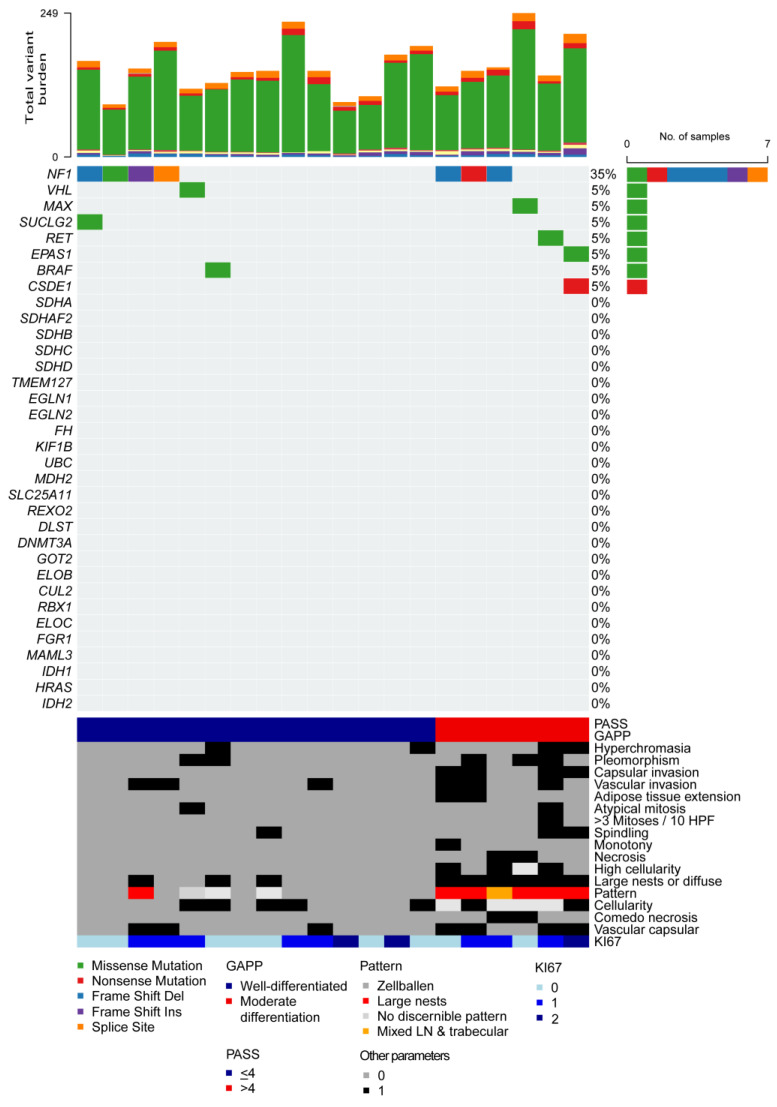
Oncoplot showing somatic variants in PPGL-related genes assessed in the current study, along with the pathological parameters that lead to the GAPP and PASS risk stratification.

**Figure 2:**
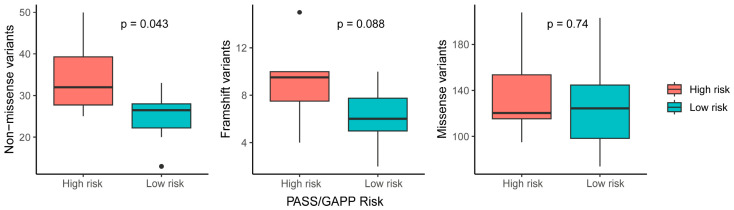
Comparison of non-missense variants, frameshift variants, and missense variants between patients with high and low risk PPGL by PASS and GAPP scores.

**Figure 3:**
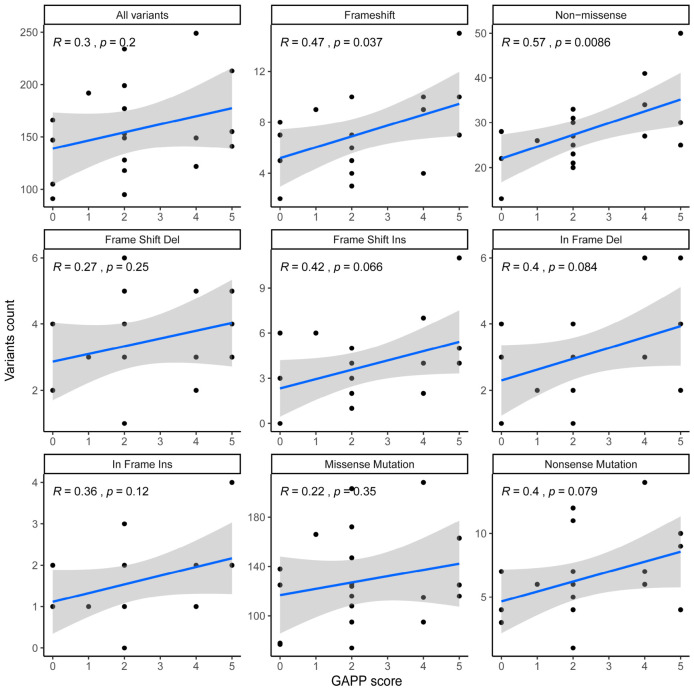
Correlation analysis between GAPP score and various variant types, showing positive correlation between PPGL aggressiveness and the number of non-missense variants.

**Figure 4:**
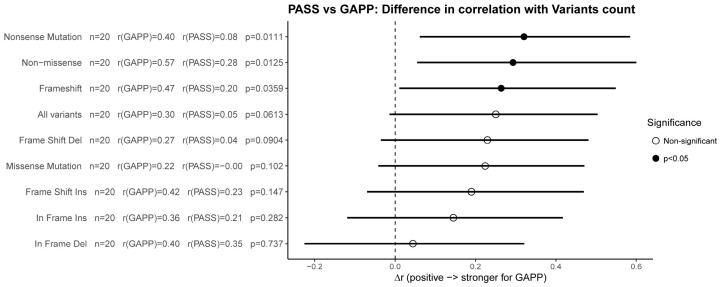
Comparison of GAPP and PASS score correlation with somatic variant load in PPGLs. The forest plot illustrates the magnitude and direction of Δr values across different variant types. A black circle indicates significant results (*p* < 0.05), an empty circle indicates non-significant results (*p* > 0.05). Δr = r(GAPP, Variants count) − r(PASS, Variants count); error bars = 95% CI.

**Figure 5:**
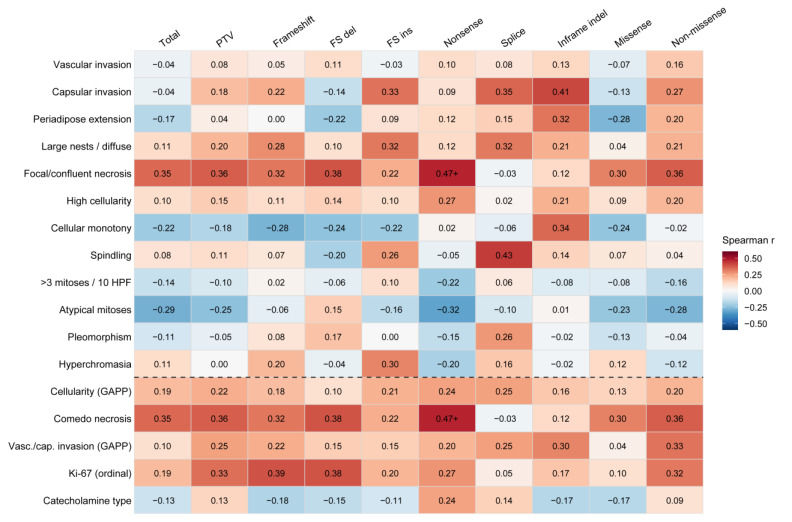
Component-wise correlation of PASS-12/GAPP-5 with somatic variant burden. Heat map of Spearman rank correlations between each PASS-12 (top block) and GAPP-5 (bottom block) histopathological component and each variant category. Cell values are r (Spearman). Within-component Benjamini–Hochberg FDR: ‘+’ marks cells with raw *p* < 0.05 that did not reach BH significance. N = 20 sporadic PPGL; putative-somatic WES.

The current study included 20 patients with PPGL. The median age at diagnosis was 49.5 years (IQR 44.0–56.3) and 14 were women (70%). Patients were divided into two groups based on their GAPP and PASS scores. Supplementary Table S2 details the pathological parameters used to define the PASS and GAPP scores as well as the comparison between the groups. The concordance rate between two independent pathologists assessing both PASS and GAPP scores was 18/20 cases (90%). Six samples were found to have a PASS score higher than 4, and the same six had a GAPP score defining them as moderately differentiated (MD). Table 1 shows the clinical parameters of our study population and the comparison between the high-risk group (PASS > 4 and MD-GAPP) and the lower-risk group. High-risk patients had larger PPGL, 4.7 cm median diameter [IQR 4.35, 6.4] vs. 3.4 cm [IQR 2.6, 4.6] than in the lower risk group (*p* = 0.03) and an approaching yet non-significant associated with increased diastolic blood pressure (90 [85, 100] vs. 79 [78, 82] mmHg, *p* = 0.09), NMN (8.5 X ULN [7.0, 9.5] vs. 2.3 X ULN [1.97, 8] *p* = 0.08), and MN (22.9 X ULN [16.81, 35.13] vs. 9.25 X ULN [4.00, 12.20] *p* = 0.099). An Oncoplot detailing the somatic variants identified in whole-exome sequencing and their relation to the pathological parameters is demonstrated in Figure 1. High-risk PPGL had a significantly higher count of non-missense variants (32.0 [27.75, 39.25] vs. 26.5 [22.25, 28], *p* = 0.04) and a non-significant association with higher rates of frameshift insertion variant count (*p* = 0.06), in-frame deletion variants (*p* = 0.09), and any frameshift indels (*p* = 0.08, Table 2 and Figure 2). A correlation analysis between GAPP score as a continuous variable and the rate of various somatic variants (Figure 3), shows a positive correlation between GAPP scores and frameshift variants (r = 0.47, *p* = 0.04) and any non-missense variants (r = 0.57, *p* = 0.009). We observed a non-significant association for frameshift insertions (*p* = 0.07), in-frame deletion (*p* = 0.08), and nonsense variants (*p* = 0.08). However, a correlation analysis between PASS score as a continuous variable and somatic variants of any type was not statistically significant (Figure 4). GAPP score demonstrated higher correlation coefficients with variant counts than the PASS score (Figure 4). Direct comparisons using Williams’ test for overlapping correlations showed that the difference between the two scores reached statistical significance for nonsense variants (*p* = 0.01), any non-missense variant (*p* = 0.01), and frameshift variants (*p* = 0.04). A further comparison of specific components of the GAPP and PASS score with somatic variant burden (Figure 5), did not demonstrate a significantly different correlation with any specific subcomponent of the GAPP and PASS score.

**Figure 6:**
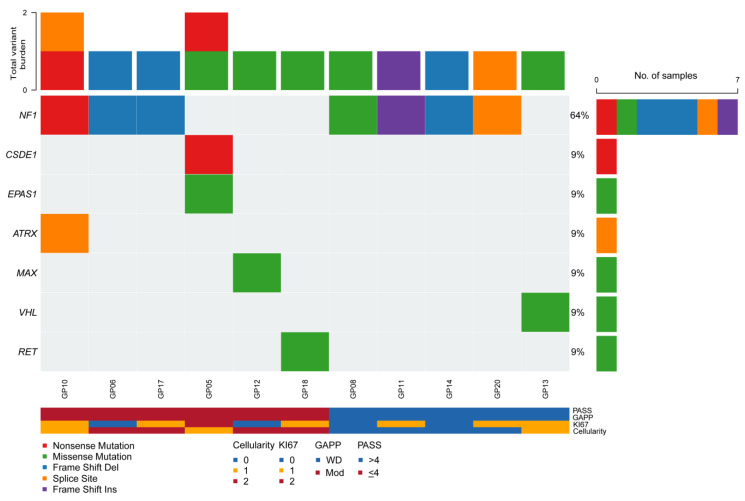
PPGL driver-gene oncoplot annotated by histopathological risk, including only canonical PPGL-related genes.

An analysis of variants exclusively associated with PPGL-driver genes demonstrated multiple variants in NF1 (Figure 6). Using a variant effect predictor demonstrated that not all truncating variants were associated with stop gain, yet even missense variants had a deleterious effect (Supplementary Table S3).

## 4. Discussion

In this pilot study, we evaluated the association between two histopathological scoring systems for PPGL and somatic genomic alterations. Overall, in our study population comprising sporadic PPGL, higher GAPP and PASS scores were associated with increased somatic variant burden of putative or enriched somatic variant set (ESVs), with a non-significant association with more nonsense mutations, in-frame insertions and frame-shift mutations. GAPP scores correlated more strongly than PASS scores with nonsense, any non-missense, and frameshift variants.

Tumor mutational burden (TMB) is associated with tumor aggressiveness across various tumor types [17]. For example, high TMB was associated with shorter progression-free and overall survival in lung adenocarcinoma [18] and with increased metastatic risk in several solid tumors [19]. TMB is relatively lower in PPGLs compared to other tumors [20]. Nevertheless, it has been reported that somatic alterations and high TMB in PPGLs correlate with more aggressive tumor behavior [21,22]. Metastatic PPGL demonstrated increased TMB compared to non-metastatic PPGL and high TMB was associated with shorter time to progression [22]. In our analysis, we chose to compare putative or enriched somatic variant set (ESVs) over TMB for several reasons. First, TMB is dominated by missense variants of uncertain significance and may not adequately distinguish biologically relevant differences between tumors. Despite this, we compared total variant counts (including missense variants), which did not differ significantly between high- and low-risk groups (*p* = 0.409), whereas non-missense (ESVs) burden did (*p* = 0.04). Second, ESVs are more likely to be functionally consequential. Frameshift, nonsense, and splice-site variants result in loss of protein function or in truncated, nonfunctional proteins, representing high-impact alterations that are more likely to disrupt tumor suppressor genes and contribute to tumor aggressiveness [23]. By focusing on ESV burden, we aimed to capture the subset of somatic alterations most likely to have biological significance in PPGL pathogenesis. ESV burden specifically captures loss-of-function events, whereas total TMB dilutes this signal with abundant passenger missense mutations. It should be noted that the higher ESVs burden associated with elevated GAPP scores does not establish biological causality, since tumor-only sequencing cannot distinguish driver from passenger events or confirm the functional consequence of individual variants. This burden should therefore be regarded as a correlate rather than a demonstrated contributor to the phenotype, and is hypothesis-generating pending validation in larger cohorts. The correlation identified in this study, showing that sporadic PPGLs with higher histopathological scores harbor more non-missense somatic alterations, is consistent with established oncologic principles. However, it remains unclear whether these increased genetic alterations drive the more aggressive histologic features or whether the two phenomena reflect a shared underlying trade-off.

Our data suggest that an increase in GAPP score, but not in PASS score, correlated with higher ESVs. Both scoring systems are used to predict the recurrence and metastatic potential of PPGLs. However, their validity varies when directly compared. While a high PASS score was found to be associated with increased risk of recurrence [24], in a meta-analysis, PASS demonstrated good positive predictive value (PPV) yet a low negative predictive value (NPV) [25]. Koh et al. demonstrated that both PASS and GAPP scores are higher in metastatic compared with non-metastatic PPGL. Yet only the GAPP score correlated with metastasis-free disease [26], especially when adding immunohistochemical staining for SDHB. A head-to-head comparison of PASS and GAPP scores, with more than five years of follow-up, identified that high GAPP scores alone, correlated with recurrence-free survival and metastatic disease [27]. Of note, in the same study the PASS scores were associated with significant inter-observer variability, demonstrating another limitation in utilizing this score. Overall, these observations suggest a potential advantage of the GAPP score over the PASS score in assessing the aggressive behavior of PPGLs.

The finding that GAPP, but not PASS, correlates with ESV burden is intriguing. Although the two scoring systems share several histopathological parameters, such as necrosis, vascular and capsular invasion, and cellularity, they differ in key conceptual aspects. The GAPP score uniquely incorporates the Ki-67 proliferation index. In our cohort, Ki-67 showed a positive but statistically non-significant directional correlation with ESVs, and no individual GAPP component independently survived correction for multiple testing. These exploratory findings are compatible with a contribution of proliferative activity to somatic-variant burden, and require validation in larger cohorts. Ki-67, a marker of cellular proliferation, has been consistently associated with tumor aggressiveness across multiple malignancies [28,29,30,31,32]. In neuroendocrine neoplasms [33], including PPGL [8], Ki-67 plays a central role in tumor grading. Specifically, in gastroenteropancreatic neuroendocrine neoplasms, classification is largely based on Ki-67 percentage and mitotic count [34,35]. Furthermore, KI-67 is associated with an increased somatic mutation rate (for example, in breast and lung cancer [36,37]). We hypothesize that Ki-67 captures proliferative and biologically active tumor behavior that may directly contribute to increased ESV burden. Higher proliferative activity may reflect enhanced tumor growth kinetics, increased metabolic demand, and potentially greater catecholamine production, all of which could contribute to a higher burden of tumor-related complications. Further insight may be obtained by examining the contribution of individual score components to ESV burden in a larger cohort.

Finally, we also observed that larger tumor size was significantly associated with higher PASS and GAPP scores. Other clinical features, including diastolic blood pressure and elevated metanephrine and normetanephrine levels showed trends toward association with higher risk histopathological scores, but these did not reach statistical significance. It has been previously reported that metastatic PPGLs are associated with larger tumor sizes [38], and that combining tumor size with PASS score [39] or CDK1 expression [40] substantially increases PPV. Although tumor size is not incorporated into either the PASS or GAPP scoring systems, our findings together with those of previous studies, support the potential utility of including tumor size in PPGL pathological risk stratification. Elevated catecholamine metabolites, particularly normetanephrine, are well established as predictive markers of PPGL aggressiveness [3,7], consistent with the trend observed in our study.

Our study has several limitations. First, it is a pilot study with a relatively small sample size and restricted score distribution; nevertheless, we detected significant differences in total variant burden between tumors with high and low histopathological scores. Second, as this was a retrospective study, we utilized archival FFPE specimens, with no paired tumor-normal analysis due to lack of available normal tissue. While this approach cannot entirely replace paired tumor-normal sequencing, it represents a validated strategy for somatic variant enrichment in tumor-only analyses where matched normal tissue is unavailable. Most but not all patients underwent formal germline genetic testing (whole-exome sequencing), yet the possibility that the germline PPGL-related variants (*NF1*, *VHL*, and *RET*) detected in the somatic tumor tissue are indeed of germline origin is low, as these gene-related syndromes are highly penetrant syndromes that the patients did not present with. Furthermore, these somatic alterations are well-recognized drivers of sporadic PPGL tumorigenesis, with *NF1* somatic alterations occurring in 40% of sporadic PPGLs [21,41]. Third, there were no outcome events (metastatic or recurrent events) in this cohort. While the median follow-up time here was over three years, only about a third of recurrent/metastatic events occur within the first 5 years of post-surgical follow-up [3]. Thus, a prolonged follow-up could possibly identify these events. Finally, the genetic sequencing approach employed was not optimized to identify large deletions or copy-number variations. Future studies should also examine the association between histopathological scoring systems and somatic variant burden in hereditary PPGL, including *SDHB* carriers, in whom tumor aggressiveness is highly variable and pathological risk stratification may provide additional clinical value.

## 5. Conclusions

This study demonstrates a significant association between histopathological scoring systems and somatic variant burden in sporadic PPGL, with the strongest signal observed for the GAPP score. These findings suggest that the GAPP scoring system may better capture the underlying genomic landscape of sporadic PPGL compared to the PASS score. Integrative approaches combining histological, molecular, biochemical, and imaging markers may be required to improve prognostication and guide clinical decision-making in PPGL. Given our study limitations, there is a need for future studies in larger, multi-center cohorts with long-term clinical outcomes to validate these results and refine integrated clinicopathological-genomic models.

## Figures and Tables

**Table 1:** Clinical characteristics of the study population.

Parameter	All	PASS > 4 GAPP MD	PASS ≤ 4 GAPP WD	*p*
n	20	6	14	
Male sex n (%)	6 (30.0)	2 (33.3)	4 (28.6)	1.000
Age at diagnosis (years)	49.50 [44.00, 56.25]	46.00 [38.75, 57.00]	50.00 [45.25, 54.50]	0.322
Follow-up time median [IQR] (months)	37.5 [21.0, 53.5]	44.0 [37.5, 49.0]	30.0 [19.0, 57.5]	0.628
Elevated MN n (%)	15 (75.0)	6 (100.0)	9 (64.3)	0.260
MN (xULN) median [IQR]	12.20 [7.30, 26.76]	22.88 [16.81, 35.13]	9.25 [4.00, 12.20]	0.099
Elevated NMN n (%)	18 (94.7)	5 (100.0)	13 (92.9)	1.000
NMN xULN median [IQR]	6.50 [2.02, 9.25]	8.52 [7.00, 9.49]	2.29 [1.97, 8.00]	0.084
HTN n (%)	12 (60.0)	5 (83.3)	7 (50.0)	0.370
Sys Max median [IQR]	140.00 [129.50, 149.50]	135.00 [127.50, 160.00]	142.00 [133.50, 149.50]	0.705
Dias Max median [IQR]	79.50 [78.25, 88.75]	90.00 [85.00, 100.00]	79.00 [78.00, 82.00]	0.085
Negative family Hx n (%)	19 (95.0)	6 (100.0)	13 (92.9)	1.000
FH PPGL syndrome n (%)	0 (0.0)	0 (0.0)	0 (0.0)	0.654
PPGL syndrome personal Hx n (%)	1 (5.0)	1 (16.7)	0 (0.0)	0.654
Max alpha blocker Doxazosin median [IQR]	4.00 [3.00, 8.00]	5.00 [2.50, 7.50]	4.00 [4.00, 8.00]	0.823
Max beta blocker Bisoprolol median [IQR]	2.50 [1.25, 5.00]	2.50 [1.25, 5.00]	2.50 [1.25, 3.12]	0.760
PPGL location right	7 (35.0)	2 (33.3)	5 (35.7)	1.000
PPGL Size cm median [IQR]	4.25 [3.08, 4.82]	4.70 [4.35, 6.40]	3.40 [2.62, 4.62]	0.032
Metastases n (%)	0 (0.0)	0 (0.0)	0 (0.0)	1.000
Recurrence n (%)	0 (0.0)	0 (0.0)	0 (0.0)	1.000

Dias—diastolic blood pressure; GAPP—grading system for adrenal pheochromocytoma and paraganglioma; HTN—hypertension; Hx—history; IQR—interquartile range; MD—moderately differentiated; MN—metanephrine; NMN—normetanephrine; PASS—pheochromocytoma of the adrenal gland scaled score; PPGL—pheochromocytoma and paraganglioma; Sys—systolic blood pressure; WD—well differentiated.

**Table 2:** High and low risk histopathological scores and types of variants.

Parameter	All	PASS > 4 GAPP MD	PASS ≤ 4 GAPP WD	*p*
FS Del median [IQR]	3.00 [2.00, 5.00]	3.50 [3.00, 4.75]	3.00 [2.00, 4.75]	0.584
FS Ins median [IQR]	3.00 [2.00, 5.00]	4.50 [4.00, 6.50]	3.00 [2.00, 3.75]	0.060
In Frame Del median [IQR]	3.00 [2.00, 4.00]	3.50 [3.00, 5.50]	3.00 [2.00, 3.00]	0.112
In Frame Ins median [IQR]	1.50 [1.00, 2.00]	2.00 [2.00, 2.00]	1.00 [1.00, 2.00]	0.089
Missense Mutation median [IQR]	124.50 [104.75, 151.00]	120.50 [115.25, 153.50]	124.50 [98.25, 144.75]	0.710
Nonsense Mutation median [IQR]	6.00 [4.00, 7.50]	8.00 [6.25, 9.75]	5.00 [4.00, 6.75]	0.095
Nonstop Mutation median [IQR]	0.00 [0.00, 0.00]	0.00 [0.00, 0.00]	0.00 [0.00, 0.00]	0.342
Splice Site median [IQR]	9.50 [8.00, 11.25]	11.00 [9.25, 13.50]	9.00 [8.00, 10.75]	0.212
Non-missense median [IQR]	27.00 [24.50, 30.25]	32.00 [27.75, 39.25]	26.50 [22.25, 28.00]	0.039
FS median [IQR]	7.00 [5.00, 9.25]	9.50 [7.50, 10.00]	6.00 [5.00, 7.75]	0.081
Total variants median [IQR]	149.00 [126.50, 180.75]	152.00 [143.00, 198.50]	149.00 [120.50, 174.25]	0.409

Del—deletion; FS—frame shift; Ins—insertion; IQR—interquartile range.

## Data Availability

The datasets used and/or analyzed during the current study are available from the corresponding author on reasonable request.

## Supplementary Materials

The following supporting information can be downloaded at: https://www.mdpi.com/article/10.3390/cancers18121983/s1.
