# Supplementary material for: The GAPP Aggressivity Score Correlates with Total Enriched Somatic Variant Burden in Sporadic Pheochromocytoma—A Pilot Study
Source: Cancers (Basel). 2026 Jun 18;18(12):1983. doi: 10.3390/cancers18121983 (PMC13296916; doi:10.3390/cancers18121983)
Supplement: Supplementary file 1 [file cancers-18-01983-s001.zip › cancers-4274951-supplementary.pdf]

Supplementary Table S1. PASS and GAPP score system. Adopted from [9, 10]

**PASS**		**GAPP**	
**Parameter**	**Score**	**Parameter**	**Score**
Large nests of diffuse growth (>10% of tumor volume)	2	Zellballen	0
Central (middle of large nests) or confluent tumor necrosis (not degenerative change)	2	Large and irregular cell nest	1
High cellularity	2	Pseudorosette (even focal)	1
Tumor cell spindling (even focal)	2	Cellularity<br>Low (<150 cells/U)<br>Moderate (150–250 cells/U)<br>High (more than 250 cells/U)	 0<br>1<br>2
Mitotic figure >3/10 HPF	2	Comedo necrosis<br>Absence<br>Presence	 0<br>2
Atypical mitotic figures	2	Vascular or capsular invasion<br>Absence<br>Presence	 0<br>1
Extension into adipose tissue	2	Ki67 labelling index (%)<br><1<br>1–3<br>>3	 0<br>1<br>2
Vascular invasion	1	Catecholamine type<br>Epinephrine type (E or E+NE)<br>Norepinephrine type (NE or NE+DA)<br>Non-functioning type	 0<br>1<br>0
Capsular invasion	1		
Profound nuclear pleomorphism	1		
Nuclear hyperchromasia	1		
**Total maximum score**	20	**Total maximum score**	10
**Benign**<br>**Aggressive**	<4<br>4≤	**Well-differentiated**<br>**Moderately differentiated**<br>**Poorly differentiated**	0-2<br>3-6<br>7-10

DA – dopamine; E – epinephrine; NE – norepinephrine,

Supplementary Table S2. Pathologic parameters and comparison according to risk of recurrence per PASS and GAPP scores.

Parameter	All	PASS >4 GAPP MD	PASS <=4 GAPP WD	p
n	20	6	14	
Hyperchromasia n(%)	4 (20.0)	2 (33.3)	2 (14.3)	0.714
Pleomorphism n(%)	5 (25.0)	3 (50.0)	2 (14.3)	0.260
Capsular invasion n(%)	4 (20.0)	4 (66.7)	0 (0.0)	0.005
Vascular invasion n(%)	6 (30.0)	3 (50.0)	3 (21.4)	0.456
Adipose tissue extension n(%)	2 (10.0)	2 (33.3)	0 (0.0)	0.143
Atypical mitosis n(%)	2 (10.0)	1 (16.7)	1 (7.1)	1.000
Mitose n(%)	1 (5.0)	1 (16.7)	0 (0.0)	0.654
Spindling n(%)	3 (15.0)	2 (33.3)	1 (7.1)	0.412
Monotony n(%)	1 (5.0)	1 (16.7)	0 (0.0)	0.654
Necrosis n(%)	2 (10.0)	2 (33.3)	0 (0.0)	0.143
High cellularty 0/1/2 n(%)	16/3/1 (80.0/15.0/5.0)	2/3/1 (33.3/50.0/16.7)	14/0/0 (100/0/0)	0.003
Large nests or diffuse n(%)	9 (45.0)	6 (100.0)	3 (21.4)	0.006
PASS median [IQR]	2.00 [0.00, 6.00]	7.00 [6.25, 9.25]	0.50 [0.00, 2.50]	<0.001
Pattern n(%)				0.003
Diffuse growth	2 (10.0)	0 (0.0)	2 (14.3)	
Large nests	6 (30.0)	5 (83.3)	1 (7.1)	
Mixed LN trabecular	1 (5.0)	1 (16.7)	0 (0.0)	
No discernible pattern	1 (5.0)	0 (0.0)	1 (7.1)	
Zellballen	10 (50.0)	0 (0.0)	10 (71.4)	
Cellularity n(%) 0/1/2	9/7/4 (45.0/35.0/20.0)	0/2/4 (0/33.3/66.7)	9/5/0 (64.3/35.7/0)	0.001
Comedo necrosis n(%)	2 (10.0)	2 (33.3)	0 (0.0)	0.143
Vascular capsular n(%)	7 (35.0)	4 (66.7)	3 (21.4)	0.152
KI67 0/1/2 n(%)	9/8/3 (45.0/40.0/15.0)	2/3/1 (33.3/50.0/16.7)	7/5/2 (50.0/35.7/14.3)	0.783
GAPP median [IQR]	2.00 [1.75, 4.00]	4.50 [4.00, 5.00]	2.00 [0.25, 2.00]	<0.001
CgA n(%)	19 (95.0)	5 (83.3)	14 (100.0)	0.654
Synaptophysin n(%)	17 (85.0)	5 (83.3)	12 (85.7)	1.000
SDHA n(%)	5 (25.0)	2 (33.3)	3 (21.4)	1.000
SDHB n(%)	9 (45.0)	2 (33.3)	7 (50.0)	0.844
S100 n(%)	15 (75.0)	4 (66.7)	11 (78.6)	1.000
Mitoses 3 n(%)	1 (5.0)	1 (16.7)	0 (0.0)	0.654
High cellularity 0/1/2 n(%)	16/3/1 (80.0/15.0/5.0)	2/3/1 (33.3/50.0/16.7)	14/0/0 (100.0/0/0)	0.003

CgA – chromogranin A; GAPP – grading system for adrenal pheochromocytoma and paraganglioma; IQR – interquartile range; MD – moderately differentiated; PASS - pheochromocytoma of the adrenal gland scaled score; WD – well differentiated

Supplementary Table S3

Sample	Gene Hugo Symbol	Chr	Start Position	Reference Allele	Tumor Sequence Allele	Variant Classification	cDNA Change	Protein Change	Tumor alternate-allele (variant) read count	Tumor reference-allele read count	VAF	VAF class	Truncating	VEP consequence	CADD	SIFT	PolyPhen	ClinVar significance	dbSNP	COSMIC
GP05	CSDE1	chr1	114734032	A	C	Nonsense	c.278T>G	p.L93*	21	27	0.4375	Clonal	Yes	stop gained	38	NA	NA	not in ClinVar	novel	
GP05	EPAS1	chr2	46376696	G	A	Missense	c.1192G>A	p.E398K	6	73	0.075949	Subclonal	No	missense variant	29.5	deleterious	NA	uncertain significance	rs1283870670	
GP06	NF1	chr17	31343024	T	-	Frame Shift Del	c.7015delT	p.F2339fs	44	32	0.578947	Clonal	Yes	frameshift variant	NA(indel)	NA	NA	not in ClinVar	novel	
GP08	NF1	chr17	31349129	A	G	Missense	c.7136A>G	p.H2379R	46	18	0.71875	Clonal	No	missense variant	25.6	Deleterious low confidence	Possibly damaging	uncertain significance/likely pathogenic	rs1597858362	COSM5944156
GP10	NF1	chr17	31206360	C	T	Nonsense	c.1381C>T	p.R461*	40	8	0.833333	Clonal	Yes	stop gained	36	NA	NA	pathogenic	rs878853865	COSM24464
GP10	ATRX	chrX	77557451	C	A	Splice Site	c.6699G>T	p.K2233N	6	31	0.162162	Subclonal	Yes	missense variant	29.9	Deleterious low confidence	NA	not in ClinVar	novel	
GP11	NF1	chr17	31200508	-	AT	Frame Shift Ins	c.975_976insAT	p.K326fs	40	20	0.666667	Clonal	Yes	frameshift variant	NA(indel)	NA	NA	not in ClinVar	novel	
GP12	MAX	chr14	65093740	G	A	Missense	c.112C>T	p.R38W	7	65	0.097222	Subclonal	No	missense variant	34	deleterious	Probably damaging	uncertain significance	rs866762160	COSM1370646
GP13	VHL	chr3	10142124	G	A	Missense	c.277G>A	p.G93S	19	125	0.131944	Subclonal	No	Missense variant	27.6	deleterious	Probably damaging	pathogenic	rs5030808	COSM18080
GP14	NF1	chr17	31235641	T	-	Frame Shift Del	c.3739delT	p.F1247fs	27	50	0.350649	Clonal	Yes	Frameshift variant	NA(indel)	NA	NA	not in ClinVar	novel	
GP17	NF1	chr17	31326217	AAGCTA GCTCACA	-	Frame Shift Del	c.5170_5182del AAGCTAGCTCACA	p.L1725fs	18	46	0.28125	Clonal	Yes	Frameshift variant	NA(indel)	NA	NA	not in ClinVar	novel	
GP18	RET	chr10	43121968	T	C	Missense	c.2753T>C	p.M918T	46	47	0.494624	Clonal	No	Missense variant	27.2	deleterious	Probably damaging	pathogenic/likely pathogenic	rs74799832	COSM965
GP20	NF1	chr17	31248982	A	G	Splice Site	c.e30-2A>G		30	24	0.555556	Clonal	Yes	Splice acceptor variant	35	NA	NA	pathogenic	rs864622431	COSM1382109

variants in PPGL-related genes.

Chr -Chromosome CADD, Combined Annotation-Dependent Depletion; cDNA, coding-DNA; COSMIC, Catalogue Of Somatic Mutations In Cancer identifier; HUGO, Human Genome Organization; PolyPhen, Polymorphism Phenotyping; SIFT, Sorting Intolerant From Tolerant; VAF, variant allele frequency; VE{, Ensembl Variant Effect Predictor

Supplementary Figure S1. Representative histopathological images. "Zellballen" architectural pattern, consisting of nests of tumor cells separated by peripheral capillaries (**A**); Diffuse growth pattern with hyaline globules (**B**), and Tubercular morphology (**C**). X 20 magnification.

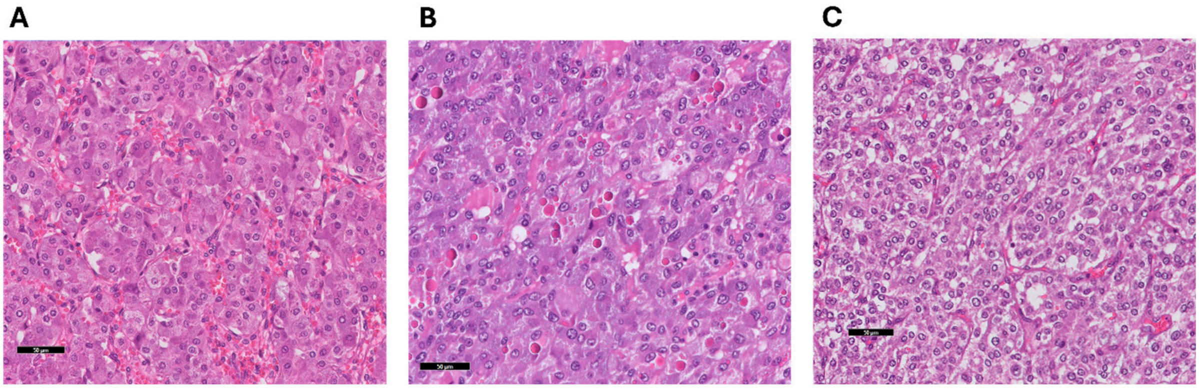

Supplementary Figure S2. Per-sample quality control and tumor purity: Target-depth coverage (percent of variant sites at <30×, 30–50×, 50–100× and ≥100× depth, **A**), tumor purity by gaussian model (**B**) and VAF distribution (red - high-risk, blue - low-risk, **C**).

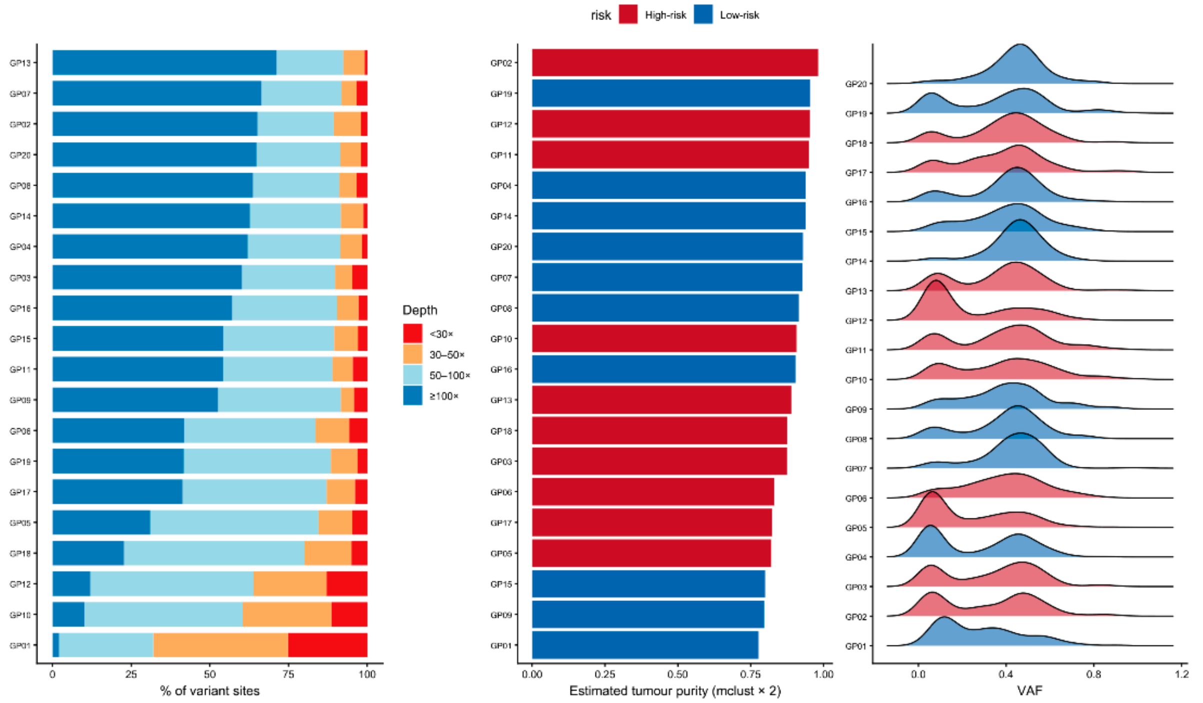

*Purity was defined as 2 × mean of the highest-VAF clonal-heterozygous component in [0.10, 0.55].

Supplementary Figure S3. C>T/G>A transition fraction as a marker for FFPE-deamination QC and its relation to PTV burden. Per sample C>T/G>A transition fraction (**A**), and its correlation with PTV burden (**B**).

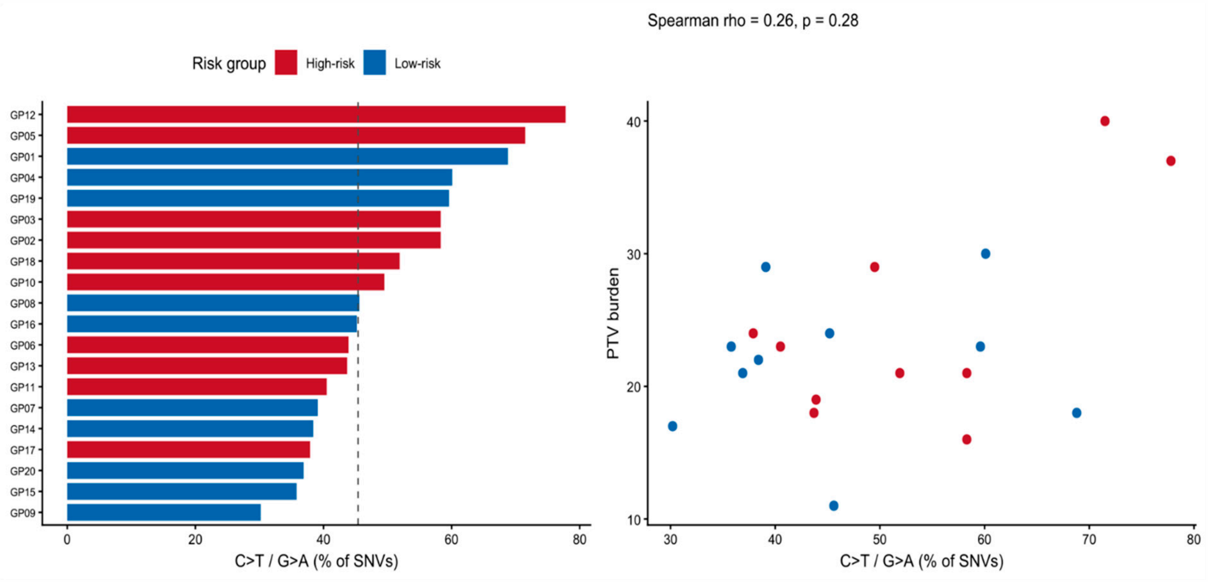

FFPE, formalin-fixed paraffin embedded.
